# Supplementary material for: Odd electron wave packets from cycloidal ultrashort laser fields
Source: Nat Commun. 2019 Feb 8;10:658. doi: 10.1038/s41467-019-08601-7 (PMC6368582; doi:10.1038/s41467-019-08601-7)
Supplement: Supplementary file 1 — Supplementary Information [file 41467_2019_8601_MOESM1_ESM.pdf]

# Odd electron wave packets from cycloidal ultrashort laser fields

Kerbstadt et al.

## SUPPLEMENTARY INFORMATION: ODD ELECTRON WAVE PACKETS FROM CYCLOIDAL ULTRASHORT LASER FIELDS

The supplementary information is subdivided in four parts. The bichromatic $4f$ white light polarization pulse shaping scheme [1–3] applied in the main paper "Odd electron wave packets from cycloidal ultrashort laser fields" features a high level of polarization control to create arbitrary cycloidal femtosecond laser fields. Thus, in the first section, we present a detailed mathematical description of the corresponding electrical laser fields, its polarization profiles and the general optical symmetry properties. Because cycloidal laser fields give rise to varied free electron wave packets (FEWPs) with arbitrary rotational symmetry from fully controlled multipath interferences, we derive a quantum mechanical description of FEWPs from bichromatic multiphoton ionization (MPI) in the second part. Furthermore, the interplay between symmetries in light and matter is discussed. In the third part, the data processing and additional phase-dependent measurements supporting the results within the main paper are presented. In general, cycloidal fields open new experimental degrees of quantum control inaccessible with ultrashort polarization-tailored single color laser pulse sequences. For example, the combination of carrier-envelope phase (CEP) control and bichromatic multipath interferences gives rise to arbitrary even- and odd-numbered rotational symmetries in matter waves. These novel features, provided by shaper-generated bichromatic carrier-envelope phase-stable polarization-tailored supercontinuum (BiCEPS) pulses, are discussed in the final fourth section.

### Supplementary Note 1: Description of the laser field

We start with a mathematical description of a commensurable ($N_1\omega$:$N_2\omega$) bichromatic laser field and its cycloidal polarization profile. The bichromatic counterrotating circularly polarized (CRCP) pulse sequence, used in our experiments, consists of an initial red, left-handed circularly polarized (LCP; $j = 1$, $q_1 = 1$, $\boldsymbol{e}_1 = -\frac{1}{\sqrt{2}}(\boldsymbol{e}_\mathrm{H} + i\boldsymbol{e}_\mathrm{V}) = -\frac{1}{\sqrt{2}}\{1, i\}$) pulse $\boldsymbol{E}_1(z,t)$ followed by a blue, right-handed circularly polarized (RCP; $j = 2$, $q_2 = -1$, $\boldsymbol{e}_{-1} = -\frac{1}{\sqrt{2}}(\boldsymbol{e}_\mathrm{H} - i\boldsymbol{e}_\mathrm{V}) = -\frac{1}{\sqrt{2}}\{1, -i\}$) pulse $\boldsymbol{E}_2(z,t)$. Here, $\boldsymbol{e}_\mathrm{H} = \{1, 0\}$ and $\boldsymbol{e}_\mathrm{V} = \{0, 1\}$ are the Jones vectors for horizontally ($-y$-direction) and vertically ($x$-direction) polarized light, respectively. The CRCP pulse sequence is described by

$$\begin{aligned} \boldsymbol{E}(z,t) &= \boldsymbol{E}_1(z,t) + \boldsymbol{E}_2(z,t) \\ \boldsymbol{E}_j(z,t) &= \boldsymbol{e}_{q_j}\mathcal{E}_j(t-\tau_j)\, e^{i[k_j z - (\omega_j t + \hat{\varphi}_j + \varphi_\mathrm{ce})]}. \end{aligned} \tag{1}$$

Both pulses ($j = 1, 2$) are described by the envelope functions $\mathcal{E}_j(t)$, the center frequencies $\omega_j = c\,k_j$, the carrier-envelope phase (CEP) $\varphi_\mathrm{ce}$ and the relative phases $\hat{\varphi}_j$. The latter $\hat{\varphi}_j = \varphi_j - \omega_j\tau_j$ include a constant phase $\varphi_j$ and an additional phase term of $-\omega_j\tau_j$, accounting for the time delay $\tau_j$. With regard to the experiments presented in the main paper and for simplicity, we restrict ourselves to the case $\tau_2 = 0$, such that $\hat{\varphi}_1 = \varphi_1 - \omega_1\tau_1$ and $\hat{\varphi}_2 = \varphi_2$. Bichromatic ($N_1\omega$:$N_2\omega$) fields have a frequency ratio of $\omega_1 : \omega_2 = N_1 : N_2$. Bichromatic corotating circularly polarized (COCP) fields feature identical polarization of both pulses, that is, either LCP/LCP or RCP/RCP.

To elucidate the influence of the optical phases on the rotation of the polarization profile of a CRCP or COCP pulse sequence, we consider the eigenvalues and eigenvectors of the rotation matrix $\boldsymbol{R}(\alpha) = \begin{pmatrix}\cos\alpha & -\sin\alpha \\ \sin\alpha & \cos\alpha\end{pmatrix}$,

$$\boldsymbol{R}(\alpha)\,\boldsymbol{e}_q = e^{-qi\alpha}\boldsymbol{e}_q, \tag{2}$$

where $\boldsymbol{e}_q$ are the Jones vectors $\boldsymbol{e}_1$ and $\boldsymbol{e}_{-1}$ for LCP and RCP light defined above. The rotation angle $\alpha$ is taken from the $x$- to the $y$-axis in $\phi$-direction. Application of the rotation matrix to a CRCP pulse sequence yields

$$\boldsymbol{R}(\alpha)\,(\boldsymbol{e}_1\mathcal{E}_\mathrm{L} + \boldsymbol{e}_{-1}\mathcal{E}_\mathrm{R}) = \boldsymbol{e}_1\mathcal{E}_\mathrm{L}e^{-i\alpha} + \boldsymbol{e}_{-1}\mathcal{E}_\mathrm{R}e^{i\alpha}. \tag{3}$$

Here, $\mathcal{E}_\mathrm{L}$ and $\mathcal{E}_\mathrm{R}$ describe the time-delayed envelopes of the LCP and RCP pulse, respectively, including all optical phases. From Supplementary Eq. (3) we see, that a rotation of the CRCP pulse sequence by an angle of $\alpha$ is equivalent to a relative phase of $2\alpha$ between the two colors. Therefore, by introducing a relative phase between the colors we rotate the entire pulse about the $z$-axis. This technique is particularly advantageous for photoelectron tomography with ultra-broadband pulses since a $\lambda/2$ wave plate is not required for pulse rotation. Applying the above definition

of the bichromatic field to the eigenvalue equation, one finds the rotation angles $\alpha_\tau^{\mathrm{cr}}$ and $\alpha_\tau^{\mathrm{co}}$

$$\begin{aligned}\alpha_\tau^{\mathrm{cr}} &= \frac{N_2 - N_1}{N_2 + N_1}\varphi_{\mathrm{ce}} + \frac{N_2}{N_2 + N_1}\hat{\varphi}_1 - \frac{N_1}{N_2 + N_1}\varphi_2\\ \alpha_\tau^{\mathrm{co}} &= \frac{N_2 - N_1}{N_2 - N_1}\varphi_{\mathrm{ce}} + \frac{N_2}{N_2 - N_1}\hat{\varphi}_1 - \frac{N_1}{N_2 - N_1}\varphi_2\end{aligned} \tag{4}$$

for CRCP and COCP bichromatic fields, respectively.

## Supplementary Note 2: Description of the free electron wave packets

FEWPs created by non-resonant $N$-photon ionization of a hydrogen-like atom, such as sodium, are composed of continuum states $\psi_{\ell,m}$, whose angular part is described by the spherical harmonics $Y_{\ell,m}(\theta,\phi)$. For later purposes, the angular part is further decomposed into a polar part $\mathcal{P}_{\ell,m}[\cos(\theta)]$, related to the associated Legendre polynomials, and the azimuthal part $e^{im\phi}$, such that

$$\psi_{\ell,m} \propto \mathcal{P}_{\ell,m}[\cos(\theta)]e^{im\phi}. \tag{5}$$

The radial part of the FEWPs is essentially determined by the $N$th-order spectrum of the ionizing laser pulse [4, 5], which introduces additional phases. To describe our experimental data, we consider bichromatic MPI by an $(N_1\omega{:}N_2\omega)$ BiCEPS pulse sequence consisting of an initial red LCP pulse ($\sigma^+$; index 1) followed by a blue LCP (COCP case) or RCP (CRCP case) pulse ($\sigma^\pm$; index 2). Ionization by the first pulse requires the absorption of $N_2$ photons. Due to the selection rules $\Delta\ell = +1$ and $\Delta m = \pm 1$ for $\sigma^\pm$ transitions, the angular momentum quantum numbers of the FEWP after interaction with the first pulse are $\ell = m = N_2$. As introduced in the previous section, the spectral phase of the first pulse is $-(\varphi_1 + \varphi_{\mathrm{ce}})$ and, hence, the phase of the $N_2$th-order spectrum is $-N_2(\varphi_1 + \varphi_{\mathrm{ce}})$. Due to its evolution during the time delay $\tau$ between the two pulses, the FEWP created by the first pulse acquires an additional energy-dependent phase of $-\varepsilon\tau/\hbar$. Moreover, $N_2$th-order time-dependent perturbation theory yields an additional phase factor of $i^{N_2}$ [4]. Therefore, the first partial electron wave packet after absorption of $N_2$ photons from the first pulse reads

$$\tilde{\psi}_{N_2,N_2} \propto \psi_{N_2,N_2}\, i^{N_2}\, e^{-iN_2(\varphi_1+\varphi_{\mathrm{ce}})}\, e^{-i\varepsilon\tau/\hbar}. \tag{6}$$

Here, the tilde is used to distinguish between the laser-excited state and the unperturbed eigenstates $\psi_{\ell,m}$. Analogously, $N_1$-photon ionization by the second blue LCP/RCP pulse with spectral phase $-(\varphi_2 + \varphi_{\mathrm{ce}})$ gives rise to the partial wave packet

$$\tilde{\psi}_{N_1,\pm N_1} \propto \psi_{N_1,\pm N_1}\, i^{N_1}\, e^{-iN_1(\varphi_2+\varphi_{\mathrm{ce}})}. \tag{7}$$

The total electron wave function is obtained by coherent superposition of both partial electron wave packets

$$\psi = \tilde{\psi}_{N_2,N_2} + \tilde{\psi}_{N_1,\pm N_1}. \tag{8}$$

In the main paper, we distinguish between three distinct cases, that is, the directional, the odd and the spiral-shaped FEWPs, corresponding to MPI with parallel linearly polarized (PLP), temporally overlapping BiCEPS and time-delayed CRCP pulse sequences, respectively. According to Supplementary Eqns. (6)-(8), specifically for $N_1 = 3$ and $N_2 = 4$, the corresponding electron wave functions read

$$\begin{aligned}\psi_{\mathrm{dir}} &\propto \psi_{3,0} + i\,\psi_{4,0}\, e^{-i\Delta\varphi},\\ \psi_{\mathrm{odd}} &\propto \psi_{3,\pm 3} + i\,\psi_{4,4}\, e^{-i\Delta\varphi},\\ \psi_{\mathrm{spi}} &\propto \psi_{3,\pm 3} + i\,\psi_{4,4}\, e^{-i\Delta\varphi}e^{-i\varepsilon\tau/\hbar},\end{aligned} \tag{9}$$

with the relative quantum phase $\Delta\varphi = N_2\varphi_1 - N_1\varphi_2 + (N_2 - N_1)\varphi_{\mathrm{ce}}$.
Next, we investigate the case of spiral-shaped FEWPs – being the most general of the three cases – in more detail. The superposition state created by an $(N_1\omega{:}N_2\omega)$ BiCEPS pulse sequence is described by

$$\begin{aligned}\psi_{\mathrm{spi}} &\propto \psi_{N_1,\pm N_1} + \psi_{N_2,N_2} i^{N_2-N_1} e^{-i\Delta\varphi}e^{-i\varepsilon\tau/\hbar}\\ &\propto (\pm 1)^{N_1}\mathcal{P}_{N_1,N_1}e^{\pm iN_1\phi} + \mathcal{P}_{N_2,N_2}e^{i(N_2\phi-\Delta\varphi-\varepsilon\tau/\hbar)}e^{i\pi(N_2-N_1)/2},\end{aligned} \tag{10}$$

where we made use of the symmetry relation $Y_{\ell,-m} = (-1)^m Y^*_{\ell,m}$. In the small angular interval centered around $\theta = \pi/2$, where the photoelectron density is localized, the polar parts $\mathcal{P}_{\ell,m}$ approximately obey the relation $\mathcal{P}_{\ell+1,\ell+1} \propto -\mathcal{P}_{\ell,\ell}$. Therefore, in the following, we suppress the polar part and concentrate on the azimuthal part of the wave function, which is most relevant to the discussion of the experimental results. By this means, we arrive at the simple expression of the final state wave function

$$\psi_{\rm spi} \propto (\pm 1)^{N_1} e^{\pm i N_1 \phi} + (-1)^{N_2 - N_1} e^{i(N_2\phi - \Delta\varphi - \varepsilon\tau/\hbar + \pi(N_2 - N_1)/2)}. \tag{11}$$

The corresponding angular part of the electron density reads

$$|\psi_{\rm spi}|^2 \propto 1 + (\pm 1)^{N_1}(-1)^{N_2 - N_1} \cos\left[(N_2 \mp N_1)\phi - \Delta\varphi - \frac{\varepsilon}{\hbar}\tau + \frac{\pi}{2}(N_2 - N_1)\right]. \tag{12}$$

Using Supplementary Eq. (4) for $\tau = 0\,$fs, leads to the short-hand notation

$$\begin{aligned}\Delta\varphi &= N_2\varphi_1 - N_1\varphi_2 + (N_2 - N_1)\varphi_{\rm ce}\\ &\equiv (N_1 + N_2)\alpha_0^{\rm cr} = (N_2 - N_1)\alpha_0^{\rm co}.\end{aligned} \tag{13}$$

Finally, setting $N_1 = 3$ and $N_2 = 4$ yields the expression for the electron density given in Eq. (4) of the main paper.

## Supplementary Note 3: Data Processing and further experimental studies

In the experiment, we perform photoelectron imaging tomography [6] to reconstruct the generated 3D photoelectron wave packets using the Fourier slice algorithm [7]. Within a tomographic measurement, the pulse is rotated by 360° about its propagation direction by rotating a $\lambda/2$ wave plate and 45 projections are detected under the angles $\phi_{\lambda/2} = 0°, .., 176°$ with an angular step size of $\Delta\phi_{\lambda/2} = 4°$. In Supplementary Fig. 1, a selection of measured projections of a photoelectron wave packet from bichromatic MPI by a ($3\omega$:$4\omega$) CRCP field with $\varphi_{\rm ce} = \varphi_1 = \varphi_2 = 0$ is presented. Due to the 7-fold rotational symmetry of the FEWP, the pronounced CEP-sensitive asymmetry within the 2D projections is inverted seven times during the tomograpic procedure, that is, for rotation angles $\phi_{\lambda/2} = n \cdot 180°/7$ ($n = 1, .., 7$). The corresponding measured projections are depicted in Supplementary Fig. 1. Since the $c_7$ symmetry manifests in the asymmetric part of the projected electron densities (PEDs), the relative differences

$$\Delta(\phi_{\lambda/2}, \varepsilon, \theta) = \frac{\mathrm{PED}(\varepsilon,\theta) - \overline{\mathrm{PED}}_{\phi_{\lambda/2}}(\varepsilon,\theta)}{\overline{\mathrm{PED}}_{\phi_{\lambda/2}}(\varepsilon,\theta)} \tag{14}$$

between each individual PED and the average $\overline{\mathrm{PED}}_{\phi_{\lambda/2}}$ over all recorded PEDs [see Supplementary Fig. 1(d)] are antisymmetrized according to

$$\Delta_{\rm a}(\varepsilon,\theta) = \frac{1}{2}\left[\Delta(\varepsilon,\theta) - \Delta(\varepsilon,-\theta)\right]. \tag{15}$$

Examples are presented in Supplementary Fig. 1(a)-(c).

In order to enhance the visibility of the rotational symmetry properties of FEWPs created by BiCEPS pulses, the salient Fourier components have been increased. Supplementary Fig. 2 presents a comparison of raw and enhanced data for representative photoelectron spectra created by BiCEPS pulses of different polarization states discussed in the paper. For the FEWP created by a temporally overlapping ($3\omega$:$4\omega$) CRCP field, the $c_7$ component was enhanced by a factor of 3 [compare Supplementary Fig. 2(a) and (b)]. In case of the FEWP from overlapping ($3\omega$:$4\omega$) COCP fields, the $c_1$ component was enhanced by a factor of 4 [compare Supplementary Fig. 2(c) and (d)]. For spiral-shaped FEWPs created by time-delayed ($3\omega$:$4\omega$) CRCP fields, the $c_7$ component was enhanced by a factor of 5 [compare Supplementary Fig. 2(e) and (f)].

To compare coherent control of bichromatic MPI via the CEP $\varphi_{\rm ce}$ and the relative phase $\varphi_2$ between both colors, we generated asymmetry maps [3] by continuous variation of the respective phases, as shown in Supplementary Fig. 3. The optical phases result in a relative quantum phase

$$\Delta\varphi = N_2\varphi_1 - N_1\varphi_2 + (N_2 - N_1)\varphi_{\rm ce} \tag{16}$$

between the interfering electron wave functions from $N_1$- and $N_2$-photon ionization with temporally overlapping bichromatic fields, derived in the previous section. To this end, we employed ($3\omega$:$4\omega$) PLP fields and measured 2D

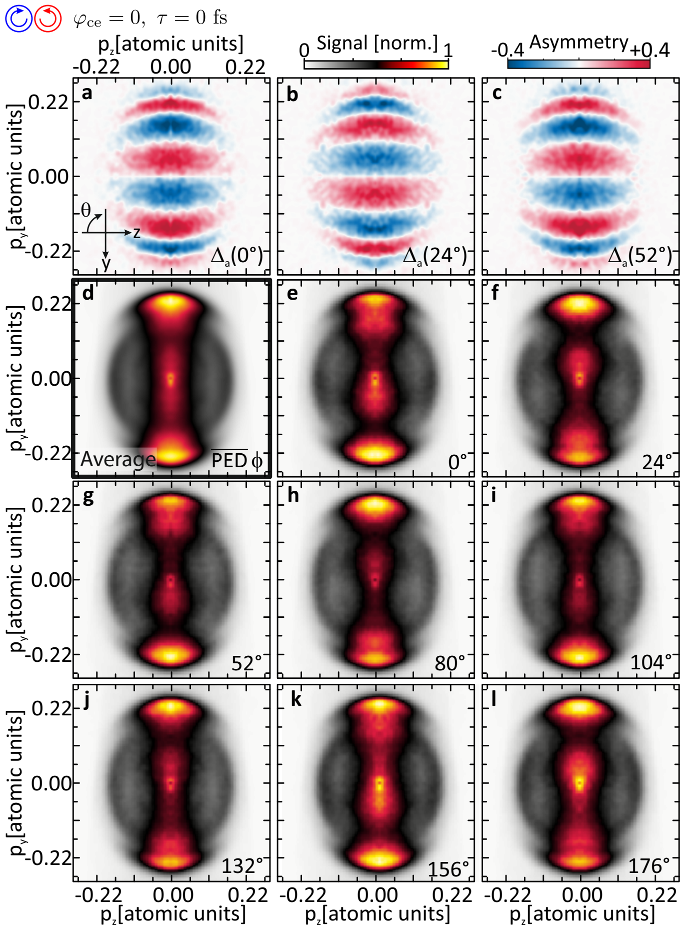


Supplementary Fig. 1. **Raw data from a tomographic measurement.** **a** - **c**, asymmetric part $\Delta_a(\phi_{\lambda/2})$ of the difference between selected PEDs in **e** - **g** and the angular average $\overline{\text{PED}}_{\phi_{\lambda/2}}$ shown in **d**. Due to the 7-fold rotational symmetry, during the photoelectron tomography where the pulse is rotated about an angle $\phi = 0°, .., 176°$, the asymmetry within the measured projections is inverted seven times. A pulse rotation about $360°/7$ leads to the same projection. Therefore, projections measured under angles of $\phi \approx n \cdot 180°/7$ ($n = 1, .., 7$) are selected and depicted in **e** - **l**. The corresponding reconstructed 3D electron wave packet is presented in the main paper.

projections of PMDs from bichromatic MPI of potassium atoms by variation of either the CEP or the absolute phase $\varphi_2$ of the blue field component. The results are presented in Supplementary Fig. 3. The measured projections are Abel-inverted and energy-calibrated in order to calculate asymmetry maps [3] with respect to the corresponding phase average. In addition, a polar representation of the energy-calibrated equatorial section through the phase-averaged photoelectron wave packet is provided for the identification of signal contributions in the asymmetry map. CEP-sensitive interferences observed in the asymmetry maps are localized in a specific kinetic energy window centered around $\varepsilon \approx 1.2$ eV. The energy window is determined by the energetically overlapping single color contributions from 3- and 4-photon ionization, leading to the interference of continuum states with opposite parity [3]. In Supplementary Figs. 3(a) and (c), asymmetry maps obtained by variation of the CEP are presented. Since the photoelectron asymmetry is inverted by CEP variation from $\varphi_{ce} = 0$ to $\varphi_{ce} = \pi$, two complete inversion cycles are observed in the interval $\varphi_{ce} \in [-2\pi, 2\pi]$. For temporally overlapping ($3\omega$:$4\omega$) fields with $\varphi_1 = \varphi_2 = 0$, the CEP-sensitive interferences are energy-independent, as shown in Supplementary Fig. 3(a).

The introduction of a time delay $\tau_1$ between the two colors by application of a linear spectral phase $\varphi_1(\omega) = \tau_1\omega$ to the red band (centered around $\omega_1$), leads to a relative quantum mechanical phase of $\Delta\varphi + \tau_1\omega$. For $\varphi_1 = \varphi_2 = 0$ and $\tau_1 = -25$ fs, an energy-dependent linear tilt with negative slope appears in the asymmetry map, as shown in Supplementary Fig. 3(c). Although the relative phase $\varphi_2$ is only varied from $-2\pi/3$ to $2\pi/3$, the respective asymmetry maps illustrated in Supplementary Figs. 3(b) and (d) also include an equivalently devolving interference pattern compared to (a) and (c) with two full inversion cycles. This is due to the fact, that $\varphi_2$ enters the quantum phase $\Delta\varphi$ with a

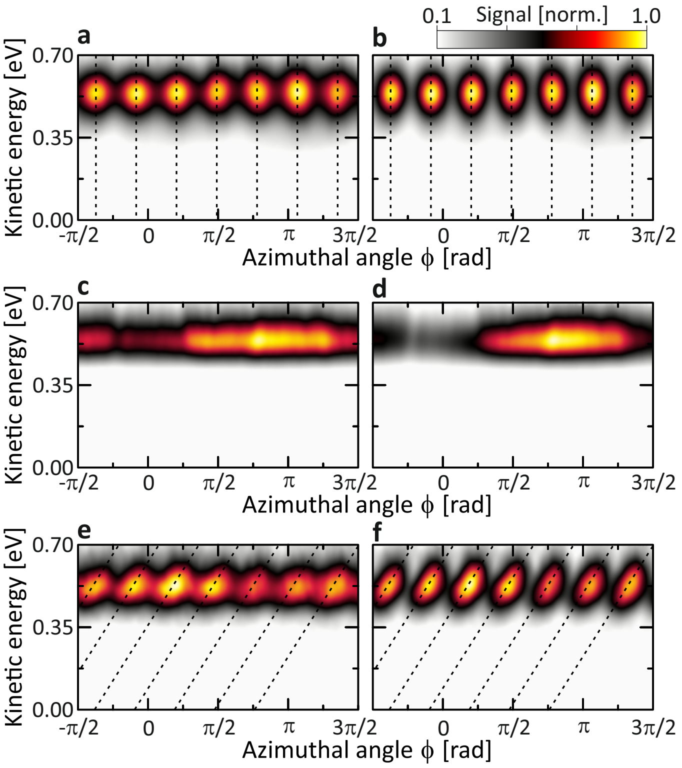


Supplementary Fig. 2. **Comparison of raw (left column) and processed (right column) experimental data. a - b**, Energy-calibrated central $x$-$y$-section of a photoelectron wave packet created by a ($3\omega$:$4\omega$) CRCP field. Raw data in **a** are compared to processed data in **b**, where the $c_7$ Fourier component was enhanced by a factor of 3. **c - d**, the same for ($3\omega$:$4\omega$) COCP fields. In this case, the $c_1$ Fourier component was enhanced by a factor of 4 and contributions from frequency mixing at lower energies are reduced. **e - f**, the same for time-delayed bichromatic ($3\omega$:$4\omega$) CRCP pulses. Here the $c_7$ Fourier component was enhanced by a factor of 5.

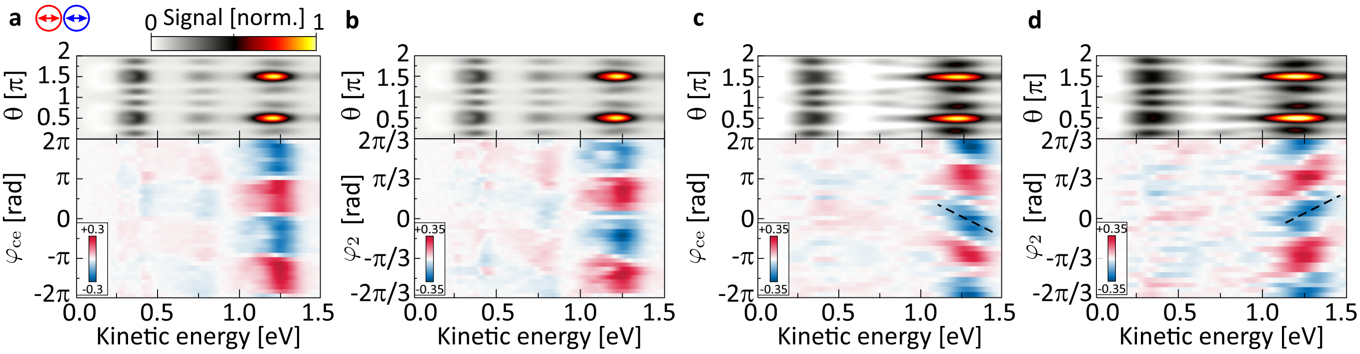


Supplementary Fig. 3. **Experimental verification of the phase relation $\Delta\varphi$ between electron wave packets.** The phases $\varphi_{\mathrm{ce}}$ and $\varphi_2$ of a ($3\omega$:$4\omega$) PLP field are varied to control the directional photoemission from potassium atoms in the MPI regime. The recorded PEDs are Abel-inverted, energy-calibrated and asymmetry maps [3] are generated from each measurement. The top frames show energy-calibrated polar representation of the $\varphi_{\mathrm{ce}}$- and $\varphi_2$-averaged equatorial section. **a** and **b**, asymmetry maps for temporally overlapping bichromatic fields measured by variation of the CEP $\varphi_{\mathrm{ce}}$ and relative phase $\varphi_2$, respectively. Phase-sensitive asymmetry oscillations are observed around $\varepsilon = 1.2\,\mathrm{eV}$, which are energy-independent. **c** and **d**, in the case of time-delayed bichromatic fields separated by $\tau_1 = -25\,\mathrm{fs}$, an energy-dependent linear tilt of the interference pattern is observed. The slope is inverted for a variation of $\varphi_{\mathrm{ce}}$ and $\varphi_2$. This verifies that both phases enter the relative phase $\Delta\varphi$ between the interfering partial electron wave packets with opposite sign.

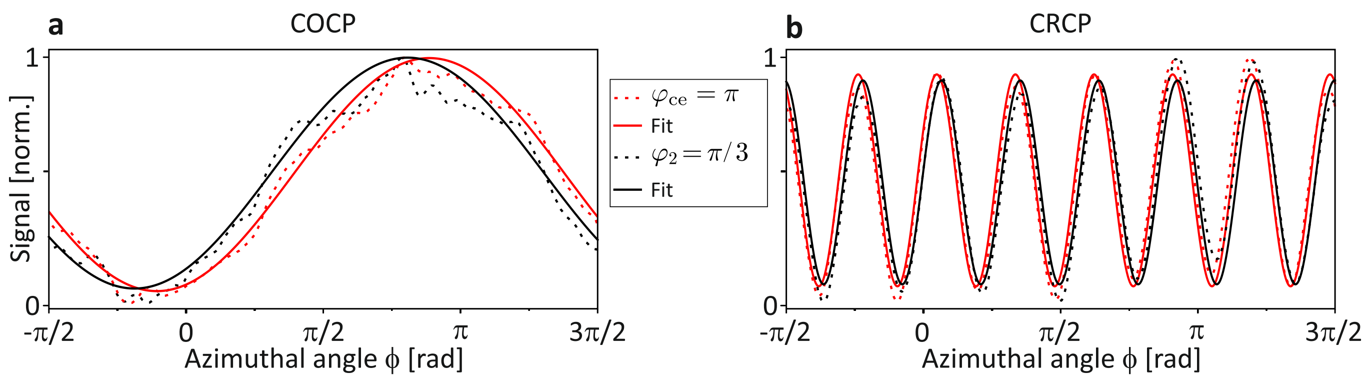


Supplementary Fig. 4. **Phase-sensitive orientations of the FEWPs from bichromatic ($3\omega$:$4\omega$) photoionization**. Angle-resolved photoelectron yield in the energy interval $\varepsilon \in [0.35\,\text{eV}, 0.70\,\text{eV}]$ of the data shown in Fig. 4 of the main paper for (a) the COCP and (b) the CRCP case. Black dashed curves correspond to $\varphi_{ce} = \pi$, red dashed curves correspond to $\varphi_2 = \pi/3$. Red and black solid lines are sinusoidal fits to the data to extract the lobe positions.

weighting factor of 3. In the case of temporally overlapping pulses, shown in Supplementary Fig. 3(b), the asymmetry map is similar to the result in (a) obtained by CEP variation. Introduction of a time delay $\tau_1 = -25\,\text{fs}$ again leads to a tilt of the interference pattern, as shown in Supplementary Fig. 3(d). However, in this case the slope is inverted compared to the CEP case in (c). The inversion of the slope serves as an experimental verification of the negative sign of the $\varphi_2$-contribution to $\Delta\varphi$ in Supplementary Eq. (16), relative to the CEP-term. As a consequence, in the case of circularly polarized bichromatic fields, the direction of rotation is opposite for the variation of $\varphi_{ce}$ and $\varphi_2$. For a detailed comparison of the phase-sensitive orientations of the the FEWPs in case of $\varphi_{ce} = \pi$ and $\varphi_2 = \pi/3$ as shown in Fig. 4 of the main paper, central sections through the retrieved 3D-FEWPs were energy-integrated over the interval $\varepsilon \in [0.35\,\text{eV}, 0.70\,\text{eV}]$), as illustrated in Supplementary Fig. 4. Slight deviations visible in the angular distributions are attributed to experimental imperfections such as fluctuations of the CEP (200 mrad longterm RMS, see Methods).

## Supplementary Note 4: Comparison of single color and bichromatic MPI

In this section, we discuss the important differences between bichromatic and single color MPI. They follow directly from the different properties of the laser fields and the ensuing laser-driven quantum dynamics. Specifically, we analyze the interplay between the symmetry properties of the light fields and the corresponding matter wave packets created by MPI. We consider three scenarios and discuss the novel features of bichromatic excitation. First, we focus on excitation with temporally overlapping single color and bichromatic CRCP and COCP pulse sequences. Then, the effect of a time delay between the field components is examined. Finally, we discuss the relevant control parameters to manipulate MPI with bichromatic and single color laser fields, respectively. In this context, we investigate the influence of phase fluctuations (relative phases and CEP) on both, the optical fields and the induced quantum dynamics. Supplementary Figure 5 provides an overview of the bichromatic and single color MPI scenarios discussed in this section. Photoelectron momentum distributions (PMD) are simulated for MPI of Na atoms with CEP-stable bichromatic ($3\omega$:$4\omega$) fields with center frequencies $\lambda_1 = 880\,\text{nm}$ and $\lambda_2 = 660\,\text{nm}$ and compared to the PMDs from single color pulses centered around $\lambda_0 = 800\,\text{nm}$.
First, we consider temporally overlapping CRCP and COCP single color and bichromatic pulse sequences and the corresponding PMD from MPI. ($N_1\omega$:$N_2\omega$) fields consist of two spectrally separated polarization-shaped bands. If both colors overlap in time, their individual polarization characteristics are imprinted in the cycloidal polarization profile of the resulting pulse. This unique feature is the basis for the generation of laser fields with unusual symmetry properties. For example, temporally overlapping CRCP bichromatic fields ($\tau = 0$) create propeller-type pulses as visualized in Supplementary Fig. 5(a) on the 7-leafed ($3\omega$:$4\omega$) CRCP pulse. The corresponding PMD reflects the 7-fold rotational symmetry of the field, as shown in the lower panel of Supplementary Fig. 5(a). In comparison, temporally overlapping single color CRCP pulse sequences are linearly polarized, as shown in Supplementary Fig. 5(e). Accordingly, 4-photon ionization results in the mirror symmetric $\psi_{4,0}$-type FEWP depicted in Supplementary Fig. 5(e). Temporally overlapping commensurable COCP bichromatic fields exhibit a heart-shaped polarization profile. The ($3\omega$:$4\omega$) COCP field shown in Supplementary Fig. 5(c) generates a crescent-shaped rotationally asymmetric FEWP. In contrast, the

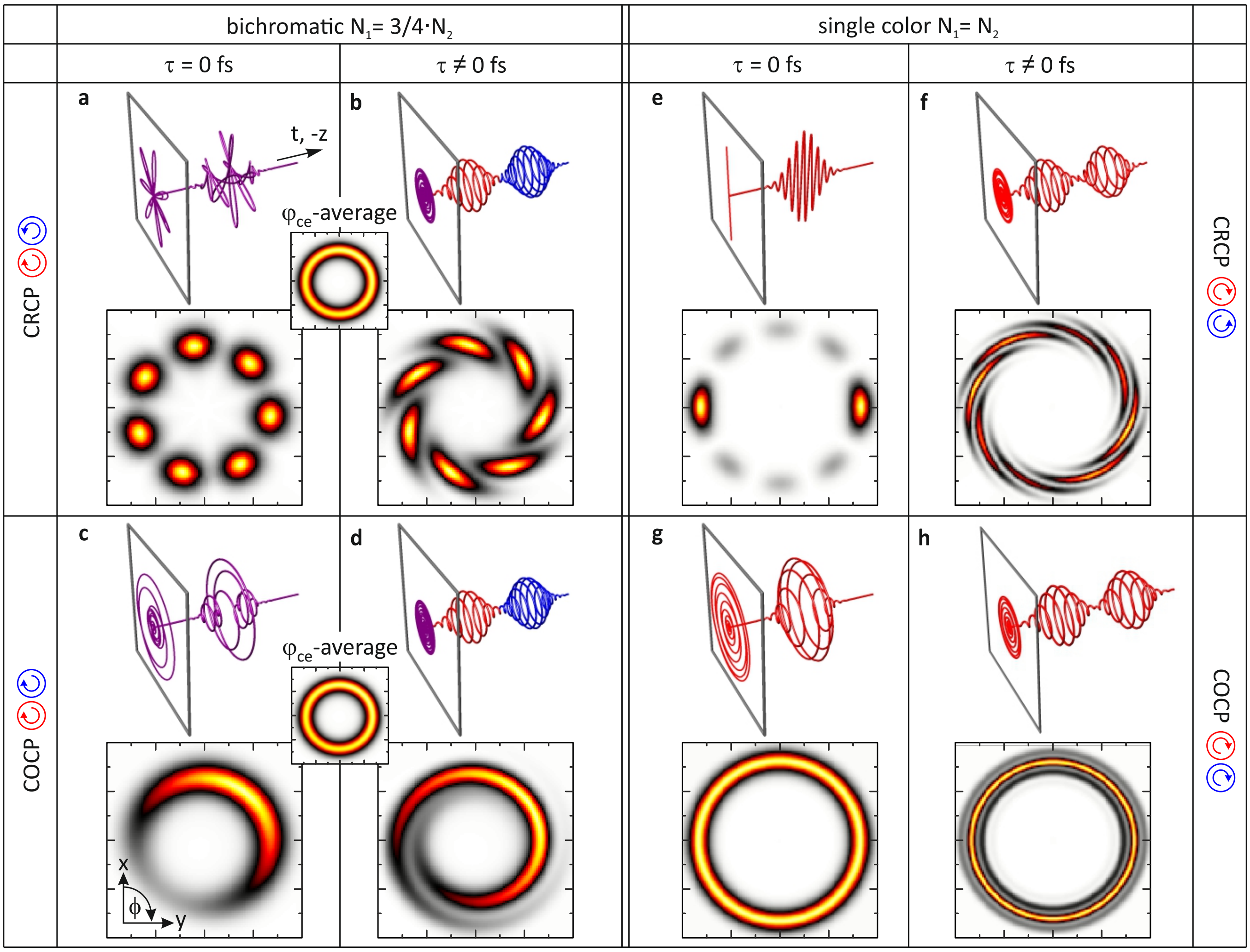


Supplementary Fig. 5. **Comparison of bichromatic and single color MPI with COCP and CRCP pulses**. Overview of numerical calculations performed to highlight the fundamental differences between CEP-stable bichromatic and single color MPI of Na atoms. Polarization-shaped laser pulses and central $x$-$y$-sections through the generated FEWPs from MPI with bichromatic ($3\omega$:$4\omega$) (left column) and single color (right column) fields are illustrated for both CRCP (top row) and COCP (bottom row) pulses ($\tau = 0$) and pulse sequences ($\tau \neq 0$). The insets in the left column display the CEP-averaged photoelectron density. In all four bichromatic scenarios, CEP-averaging results in the same torus-shaped (isotropic) angular distribution, as shown in the insets to (a)-(d). In (a)-(b): Bichromatic ($3\omega$:$4\omega$) CRCP pulse and the respective FEWP with 7-fold rotational symmetry for $\tau = 0\,$fs (a) and a 7-armed spiral-shaped FEWP for $\tau \neq 0\,$fs (b). In (c)-(d): Bichromatic ($3\omega$:$4\omega$) COCP pulse and the respective crescent-shaped FEWP for $\tau = 0\,$fs (c) and a 1-armed spiral-shaped FEWP for $\tau \neq 0\,$fs (d). In (e)-(f): Single color CRCP pulse and the respective $g$-type FEWP for $\tau = 0\,$fs (e) and an 8-armed spiral-shaped FEWP for $\tau \neq 0\,$fs (f). In (g)-(h): Single color COCP pulse and the respective torus-shaped FEWP for $\tau = 0\,$fs (g) and a FEWP with with spectral interference fringes only in radial direction for $\tau \neq 0\,$fs (h).

polarization profile of COCP single color pulse sequences is circularly symmetric at $\tau = 0$. The corresponding FEWPs are torus-shaped and completely isotropic in azimuthal direction, as seen in Supplementary Fig. 5(g). These examples underline that bichromatic fields with cycloidal polarization profiles allow us to prepare and manipulate FEWPs inaccessible with CRCP and COCP single color fields. In particular, ($N_1\omega$:$N_2\omega$) pulses enable the creation of FEWPs with odd-numbered rotational symmetry.

Now, we discuss the influence of the time delay $\tau$ on the polarization profiles and the generated FEWPs from MPI with CRCP and COCP single color and bichromatic pulse sequences. Single color CRCP sequences with $\tau \neq 0$ have been used to create even-numbered free electron vortices [8, 9], as exemplified on the 8-armed photoelectron distribution from 4-photon ionization of Na atoms illustrated in Supplementary Fig. 5(f). The FEWP created by time-delayed single color COCP sequences is again torus-shaped, that is, fully isotropic in azimuthal direction, but modulated in

radial direction by spectral interference fringes, as shown in Supplementary Fig. 5(h). The results from time-delayed bichromatic ($3\omega$:$4\omega$) CRCP and COCP fields are depicted in Supplementary Figs. 5(b) and (d). MPI by time-delayed ($3\omega$:$4\omega$) CRCP fields gives rise to a 7-armed spiral-shaped FEWP. When both colors are fully separated in time, the polarization profile of the field becomes circularly symmetric. However, the FEWP from 3- vs. 4-photon ionization retains its 7-fold rotational symmetry. Time-delayed ($3\omega$:$4\omega$) COCP fields create a 1-armed spiral-shaped FEWP with pronounced asymmetry in azimuthal direction [see Supplementary Fig. 3(d)]. In general, FEWPs created by ($N_1\omega$:$N_2\omega$) CRCP and COCP fields exhibit arbitrary even- or odd-numbered rotational symmetry, determined by the center frequencies ratio, whereas single color MPI always creates FEWPs with even-numbered rotational symmetry. Finally, we analyze the relevant control parameters for single color and bichromatic CRCP and COCP pulse sequences. Single color MPI with phase-locked COCP and CRCP pulses is CEP-independent and depends only on the relative phase between the two driving pulses. Variations of the CEP cancel since all multiphoton pathways contribute with the same CEP. As a consequence, no CEP stabilization of the laser pulses was required for the experimental demonstration of single color electron vortices [9]. In contrast, superposition states from bichromatic $N_1$- vs. $N_2$-photon interference are sensitive to both the CEP and the relative phase between the two colors. Supplementary Eq. (4) describes the CEP- and relative phase-dependent rotation of bichromatic CRCP and COCP fields. In this case, phase fluctuations lead to a random orientation of the polarization-shaped pulse, implying that phase-sensitive quantum interferences average out. As a consequence, the CEP-averaged FEWPs from bichromatic MPI are completely symmetric − irrespective of the polarization state (CRCP or COCP) and time delay (see insets in Supplementary Fig. 5). Due to the CEP sensitivity of the shaper-generated BiCEPS fields bichromatic control experiments are much more demanding but provide refined options for phase control of the coherent electron dynamics. In this paper, we demonstrated the creation of matter waves with exceptional symmetry properties as an example of unprecedented control attained by tailored BiCEPS fields.

## Supplementary References


[1] Kerbstadt, S., Englert, L., Bayer, T. & Wollenhaupt, M. Ultrashort polarization-tailored bichromatic fields. *J. Mod. Opt.* **64**, 1010 (2017).

[2] Kerbstadt, S., Timmer, D., Englert, L., Bayer, T. & Wollenhaupt, M. Ultrashort polarization-tailored bichromatic fields from a cep-stable white light supercontinuum. *Opt. Express* **25**, 12518 (2017).

[3] Kerbstadt, S., Pengel, D., Englert, L., Bayer, T. & Wollenhaupt, M. Carrier-envelope-phase control of asymmetries in the multiphoton ionization of xenon atoms by ultrashort bichromatic fields. *Phys. Rev. A* **97**, 063402 (2018).

[4] Dudovich, N., Polack, T., Peër, A. & Silberberg, Y. Simple route to strong-field coherent control. *Phys. Rev. Lett.* **94**, 083002 –083002–4 (2005).

[5] Kerbstadt, S. *et al.* Control of photoelectron momentum distributions by bichromatic polarization-shaped laser fields. *New J. Phys.* **19**, 103017 (2017).

[6] Wollenhaupt, M. *et al.* Three-dimensional tomographic reconstruction of ultrashort free electron wave packets. *Appl. Phys. B* **95**, 647–651 (2009).

[7] Kak, A. C. & Slaney, M. *Principles of Computerized Tomographic Imaging* (IEEE Press, New York, 1988).

[8] Pengel, D., Kerbstadt, S., Englert, L., Bayer, T. & Wollenhaupt, M. Control of three-dimensional electron vortices from femtosecond multiphoton ionization. *Phys. Rev. A* **96**, 043426 (2017).

[9] Pengel, D. *et al.* Electron vortices in femtosecond multiphoton ionization. *Phys. Rev. Lett.* **118**, 053003 (2017).
